# Longitudinal remodeling of brain metastasis resection cavities after adjuvant gamma knife radiosurgery

**DOI:** 10.1007/s11060-026-05567-7

**Published:** 2026-04-28

**Authors:** Victor Goulenko, Sarthak Sinha, Babar Gulzar, Venkatesh Shankar Madhugiri, Neil D. Almeida, Lindsay Lipinski, Andrew J. Fabiano, Kenneth V. Snyder, Robert A. Fenstermaker, Robert J. Plunkett, Dheerendra Prasad

**Affiliations:** 1 Department of Radiation Medicine, Roswell Park Comprehensive Cancer Center, Elm and Carlton Streets, Buffalo, NY 14203 USA; 2 Department of Neurosurgery, Roswell Park Comprehensive Cancer Center, Buffalo, NY USA; 3 Department of Neurosurgery, Jacobs School of Medicine, Buffalo, NY USA; 4 Department of Radiation Oncology, Jacobs School of Medicine, Buffalo, NY USA

**Keywords:** Surgical cavity, Brain metastasis, Longitudinal remodeling, Stereotactic radiosurgery

## Abstract

**Purpose:**

Postoperative Gamma Knife Radiosurgery (GKRS) to resection cavities is standard for intracranial metastases, but the dynamics of these cavities after treatment and their relationship to local control remain incompletely defined.

**Methods:**

We retrospectively reviewed 98 patients with 98 resection cavities treated with single- or hypofractionated Gamma Knife radiosurgery (GKRS) between 2011 and 2025. Cavity volumes were segmented from serial contrast-enhanced MRI and compared with GKRS planning volumes. Longitudinal changes in log-transformed volumes were modeled using linear mixed-effects regression. Local recurrence–free survival (LRFS) and overall survival (OS) were estimated using Kaplan–Meier analysis, and associations with outcomes were tested using Cox proportional hazards models.

**Results:**

Median imaging follow-up was 9.1 months (IQR 4.9–16.5), and cavities showed significant volume reduction over time (β = −0.031 ln[cc]/month, *p* < 0.001). Modeling indicated a nonlinear pattern, with greater reduction early and relative stabilization thereafter. At 12 and 24 months, actuarial local control was 74.5% and 62.5%, respectively; median LRFS was 49.8 months. Volume regression occurred in both recurrent and non-recurrent groups, and the interaction between recurrence status and time was nonsignificant. Recurrence rates did not differ between single- and hypofractionated GKRS across preoperative volume strata. Higher Karnofsky performance status at surgery and at GKRS correlated with improved OS and smaller cavity volumes.

**Conclusions:**

Cavities treated with GKRS showed consistent involution over time, most pronounced early after treatment. Volumetric change alone was not associated with local control; interpretation of local failure requires dosimetric and target-definition context.

**Supplementary Information:**

The online version contains supplementary material available at 10.1007/s11060-026-05567-7.

## Introduction

Metastatic spread of cancer to the brain is common and has historically been associated with a poor prognosis [1–3]. Whole brain radiotherapy (WBRT), often with surgical resection of dominant symptomatic lesions, was long a cornerstone of management; however, WBRT is associated with neurocognitive decline that can significantly impact quality of life [4–6]. 

Over the last decade, treatment paradigms have shifted toward focal strategies that preserve cognition while maintaining oncologic outcomes [7, 8]. Maximal safe resection guided by modern imaging, followed by stereotactic radiosurgery (SRS), is now widely used for resected brain metastases [8–10]. On the Gamma Knife^®^ (GKRS; Elekta AB, Stockholm, Sweden) platform, both single-fraction GKRS (sfGKRS) and hypofractionated GKRS (hfGKRS) enable highly conformal dose delivery to postoperative cavities while sparing uninvolved brain, with survival outcomes comparable to WBRT, which is increasingly reserved for select or palliative settings [4, 7, 8, 11]. 

With improving systemic therapy and longer survival, durable intracranial control and preservation of neurologic function have become central goals [8]. Postoperative cavity-directed GKRS is widely used to provide focal adjuvant control while avoiding the neurocognitive toxicity of WBRT, and has demonstrated improved local control compared with observation after resection [7, 8, 10]. However, the resection cavity is a dynamic target [12, 13]. Cavity geometry can evolve between surgery and GKRS due to postoperative brain re-expansion and dural settling, and may be influenced by residual disease, the interval from surgery to GKRS planning, histology/biologic behavior, and concurrent systemic therapies. The postoperative cavity size is variable, and its evolution after irradiation remains an area of active investigation. Although many studies suggest a general trend toward cavity volume reduction over time, the trajectory of change (including potential nonlinearity), its relationship to local failure, and its interaction with fractionation strategy remain poorly characterized.

In this retrospective study, we evaluate longitudinal changes in postoperative cavity volume following adjuvant sfGKRS or hfGKRS delivered on the Gamma Knife^®^ platform at a single tertiary center and assess how cavity dynamics relate to local control and clinically relevant outcomes.

## Methods

### Study design and cohort

This retrospective cohort study was approved by the Institutional Review Board at Roswell Park Comprehensive Cancer Center. Consecutive patients treated between May 2011 and April 2025 who underwent surgical resection of brain metastasis followed by adjuvant GKRS to the postoperative cavity were identified.

A total of 182 patients were screened. Patients were excluded for insufficient post-GKRS imaging follow-up. The final analytic cohort comprised 98 patients (52 male, 46 female) with 98 resection cavities. Because some patients contributed more than one resection cavity, patient-level survival analyses were performed using a single index lesion per patient. For these analyses, the largest cavity volume at GKRS planning (V_GK_) was selected as the representative lesion to avoid within-patient correlation.

### Clinical and pathologic variables

Demographic and clinical variables were abstracted from the electronic medical record, including age, sex, primary tumor site and histology, and performance status. Karnofsky Performance Status (KPS) and Eastern Cooperative Oncology Group (ECOG) scores were recorded at prespecified timepoints (perioperative, at GKRS, and at recurrence or last follow-up, when available). Histological confirmation of metastatic disease was based on pathology from the primary tumor and/or an intracranial specimen. Systemic therapy exposure during follow-up could not be reliably abstracted for the entire retrospective cohort and is addressed as a limitation.

Time from surgery to GKRS was defined as the number of days between the date of craniotomy and the date of GKRS. This variable was summarized descriptively.

### GKRS treatment and planning

GKRS was delivered using different platforms (Model C^®^, Perfexion^®^, Icon^®^, or Esprit^®^; Elekta AB, Stockholm, Sweden) over time. All immobilizations were frame-based prior to Icon^®^ adoption and frame-based or mask-based thereafter, selected based on clinical and technical considerations.

All patients underwent same-day contrast-enhanced MRI for radiosurgical planning. The treatment target included the resection cavity, with adjacent dura included when clinically indicated; for deep lesions without direct dural involvement, the cavity alone was contoured. When present, synchronous unresected brain metastases were treated during the same GKRS session. No additional margins were added since no movement shift was expected on the frame-based treatments, and all patients who had thermoplastic mask fixation were continuously monitored via high-definition motion management under a threshold of 0.5 to 1 mm.

Fractionation (sfGKRS vs. hfGKRS) was individualized based on cavity size, geometry, surrounding edema, anatomic location, patient tolerance, and proximity to organs at risk (OAR), such as the optic apparatus and the brainstem.

Prescription dose (Gy), number of fractions, prescription isodose line (%), target coverage, and plan quality indices (conformity index and gradient index) were extracted from GammaPlan ^®^ treatment record. Hypofractionated regimens were predominantly delivered in three fractions.

### Imaging acquisition, segmentation, and volumetric definitions

All imaging was reviewed and segmented using BrainLab^®^ Elements SmartBrush (BrainLab, Munich, Germany) on T1-weighted, contrast-enhanced MRI sequences.

Volumes were defined as follows: Preoperative tumor volume - contoured from diagnostic or immediate preoperative MRI (used descriptively and not combined with postoperative cavity kinetics); Early postoperative cavity volume - contoured on contrast-enhanced MRI obtained within 72 h after resection, when available; GKRS baseline volume (V_GK_) was defined on the GKRS planning MRI as the prescription target volume. When adjacent dura was intentionally included in the treated target, the dural inclusion was included in V_GK_. This served as the primary baseline for longitudinal analyses. Follow-up cavity volumes (V_t_) - contoured at each post-GKRS follow-up MRI using the same segmentation approach.

Percent cavity volume change at follow-up was calculated relative to the GKRS baseline as:

$$\Delta V\left(\% \right) = \left({{V_{GK}} - {V_t}} \right)/{V_{GK}}$$

such that positive values indicate cavity involution (volume reduction) and negative values indicate cavity expansion.

Each cavity was tracked as an independent analytical unit. For patients with multiple resections via separate craniotomies, cavities were analyzed separately; when adjacent lesions were resected through a single craniotomy, resulting in a shared cavity, the combined cavity volume was analyzed as a single unit.

### Follow-up schedule and imaging windows

Follow-up MRI was performed per institutional practice (typically an early post-treatment scan followed by serial surveillance). For transparency regarding longitudinal coverage and attrition, imaging availability was summarized within prespecified post-GKRS time windows: 0–3, 3–6, 6–12, 12–24, and > 24 months. When multiple MRIs of a cavity occurred within a given window, the earliest MRI in that window was used for window-based summaries and figures.

### Outcomes and definitions

Local recurrence was assessed at the cavity level and defined as progressive or new nodular enhancement within or immediately adjacent to the treated resection cavity on serial contrast-enhanced MRI. When imaging findings were equivocal, Contrast Clearance Analysis (CCA; BrainLab, Munich, Germany) [14, 15] was used, when available, to aid adjudication of recurrence versus treatment effect; the adjudication modality (standard MRI vs. CCA) was recorded. Time to local recurrence was measured from the date of GKRS to the first imaging date meeting recurrence criteria.

Local recurrence–free survival (LRFS) was defined as the time from GKRS to local recurrence. Cavities without documented local recurrence were censored at the date of the last available intracranial imaging follow-up. Cavities from patients who died without documented local recurrence were censored at the date of death. Cavities were excluded from local control analyses only if both recurrence status and censoring/event time could not be determined.

Leptomeningeal Disease (LMD) was defined by radiographic MRI findings consistent with leptomeningeal dissemination and/or positive cerebrospinal fluid (CSF) cytology when available; time to LMD was measured from GKRS to the first diagnosis.

### Statistical analysis

Because cavity volumes were essentially right-skewed, we log-transformed them (natural log) for modeling and visualization. Log values can be negative when the volume is < 1 cc, and we excluded any measurements with volume ≤ 0 cc because they reflect segmentation or data artifacts.

Cavity volume trajectories were analyzed using a linear mixed-effects model with subject-level random effects and a first-order autoregressive (AR(1)) covariance structure, which assumes measurements taken closer together in time are more strongly correlated than those farther apart, to account for within-patient serial correlation; time was modeled as a fixed effect. Longitudinal models were fit by maximum likelihood, allowing unbalanced follow-up under a missing-at-random assumption conditional on the observed data; no imputation was performed. Imaging availability by follow-up is summarized in Supplementary Tables S1–S2.

Distributional summaries over time windows were visualized using box-and-whisker plots (median, IQR, and 1.5×IQR whiskers). Time-to-event outcomes (e.g., local control) were estimated using Kaplan–Meier methods as appropriate. For families of simultaneous comparisons (e.g., histology subgroup tests and timepoint-wise post hoc contrasts), p-values were adjusted using Benjamini–Hochberg false discovery rate control (q < 0.05). Analyses were performed in IBM SPSS Statistics^®^ (v28.0.1). The treatment era was defined a priori by GKRS year (2011–2015, 2016–2020, 2021–2025) and included as a covariate in sensitivity Cox models for LRFS and OS. Statistical significance was defined as a p-value < 0.05.

## Results

**Fig. 1:**
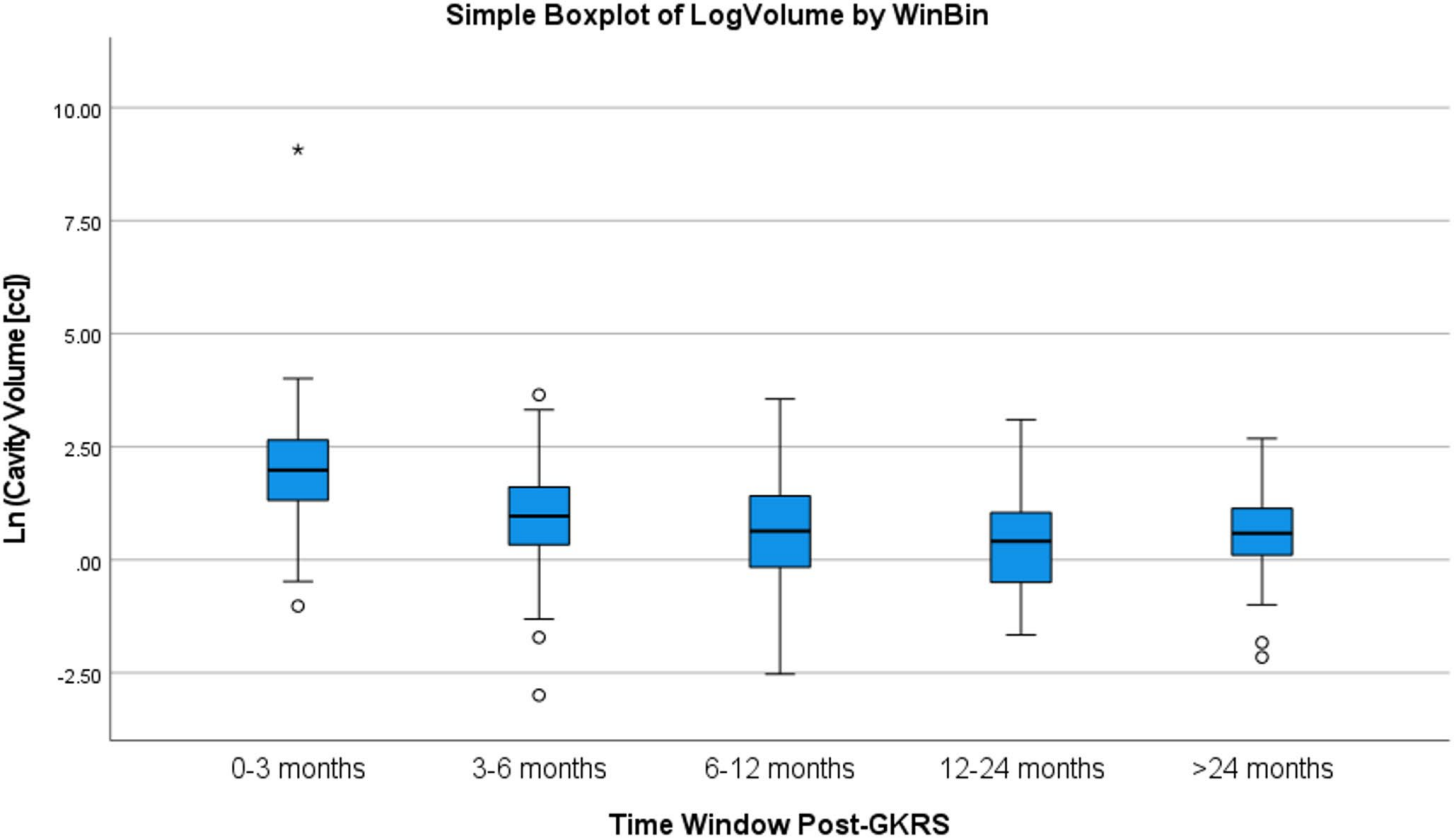
Distribution of cavity volumes by post-GKRS imaging window. Box-and-whisker plots show natural log–transformed cavity volume (ln[cc]) within prespecified post-GKRS imaging windows (0–3, 3–6, 6–12, 12–24, and > 24 months). Boxes represent the interquartile range, with the median shown as the center line; whiskers extend to 1.5×IQR, and points denote outliers. Sample sizes per window were 102, 68, 63, 32, and 15 cavities, respectively; counts are not mutually exclusive because individual cavities may contribute to multiple windows. Asterisk denotes outliers/significant contrasts

Ninety-eight patients (52 male, 46 female) with 98 resected brain metastasis cavities treated with adjuvant GKRS were included. Median imaging follow-up was 9.1 months (IQR 4.9–16.5), and five patients developed LMD following GKRS (disseminated leptomeningeal dissemination). The median time from surgery to GKRS was 20 days (IQR 13–30). Radiographic surveillance was most dense early after GKRS and declined over time: within prespecified post-GKRS windows, at least one MRI was available for 102 cavity-observations at 0–3 months, 68 at 3–6 months, 63 at 6–12 months, 32 at 12–24 months, and 15 beyond 24 months (Supplementary Table S1). Counts are not mutually exclusive because individual cavities could contribute observations to multiple windows. The distribution of ln (natural logarithm)-transformed cavity volumes by window is shown in Fig. 1.

### GKRS planning target volume and dosimetry

GKRS planning target volume and dosimetric parameters are summarized in Table 1. Unsurprisingly, hypofractionation was preferentially used for larger targets: hfGKRS cases had larger VGK and larger prescription isodose volumes (PIV) compared with sfGKRS (both *p* < 0.01). hfGKRS was also associated with higher prescribed dose and higher Dmax and Dmean (all *p* < 0.001), consistent with clinical selection of hypofractionation for larger and/or more complex cavities. Prescription isodose line, target coverage, and plan indices (PCI and GI; available cases) did not differ significantly between fractionation groups. As expected, PIV generally exceeded VGK; cases with PIV < VGK reflected less-than-complete target coverage.

**Table 1:** Gamma Knife Radiosurgery (GKRS) planning target volume and dosimetry characteristics by fractionation strategy. Values are reported as median (interquartile range) with available-case denominators. V_GK_ denotes the contoured GKRS planning target volume on the stereotactic planning MRI and is the baseline volume used for longitudinal volumetric analyses. PIV denotes the prescription isodose volume (V100%) exported from GammaPlan. PIV may be smaller than V_GK_ when target coverage is < 100%. sfGKRS indicates single-fraction Gamma Knife radiosurgery; hfGKRS indicates hypofractionated Gamma Knife radiosurgery. Comparisons between sfGKRS and hfGKRS used Mann–Whitney U tests for continuous variables and χ²/Fisher’s exact tests for categorical variables. Single-fraction GKRS (sfGKRS); hypofractionated GKRS (hfGKRS)

Parameter	Overall (*N* = 98)	sfGKRS (*N* = 74)	hfGKRS (*N* = 24)	*p*-value
V_GK_ contoured target volume (cc)	7.08 (3.72–13.89) [98/98]	6.15 (3.37–11.80) [74/74]	14.20 (6.00–24.80) [24/24]	0.002
PIV (prescription isodose volume, cc)	8.28 (5.16–16.07) [96/98]	7.33 (4.70–13.11) [73/74]	18.79 (8.22–27.95) [23/24]	< 0.001
PIV/V_GK_ ratio	1.13 (1.00–1.52) [96/98]	1.11 (1.00–1.52) [73/74]	1.22 (1.00–1.44) [23/24]	0.833
Prescribed dose (Gy)	18.00 (18.00–18.00) [98/98]	18.00 (18.00–18.00) [74/74]	22.50 (18.00–26.25) [24/24]	< 0.001
Prescription isodose line (%)	51.00 (48.00–55.00) [96/98]	51.00 (48.25–55.00) [74/74]	50.00 (47.25–54.00) [22/24]	0.439
Dmin (Gy)	13.30 (11.25–15.00) [91/98]	13.30 (11.30–14.93) [72/74]	13.20 (11.00–18.90) [19/24]	0.841
Dmean (Gy)	23.90 (22.70–25.75) [91/98]	23.60 (22.38–25.00) [72/74]	27.90 (24.35–31.00) [19/24]	< 0.001
Dmax (Gy)	35.45 (32.70–40.08) [98/98]	34.95 (31.15–36.40) [74/74]	40.75 (35.73–49.77) [24/24]	< 0.001
Coverage	0.98 (0.97–0.99) [91/98]	0.98 (0.97–0.99) [70/74]	0.98 (0.96–0.99) [21/24]	0.767
PCI	0.77 (0.68–0.81) [91/98]	0.76 (0.68–0.80) [70/74]	0.78 (0.75–0.82) [21/24]	0.313
GI	2.97 (2.77–3.13) [53/98]	2.96 (2.85–3.08) [44/74]	3.12 (2.71–3.27) [9/24]	0.758

### Longitudinal cavity volume response after GKRS

**Fig. 2:**
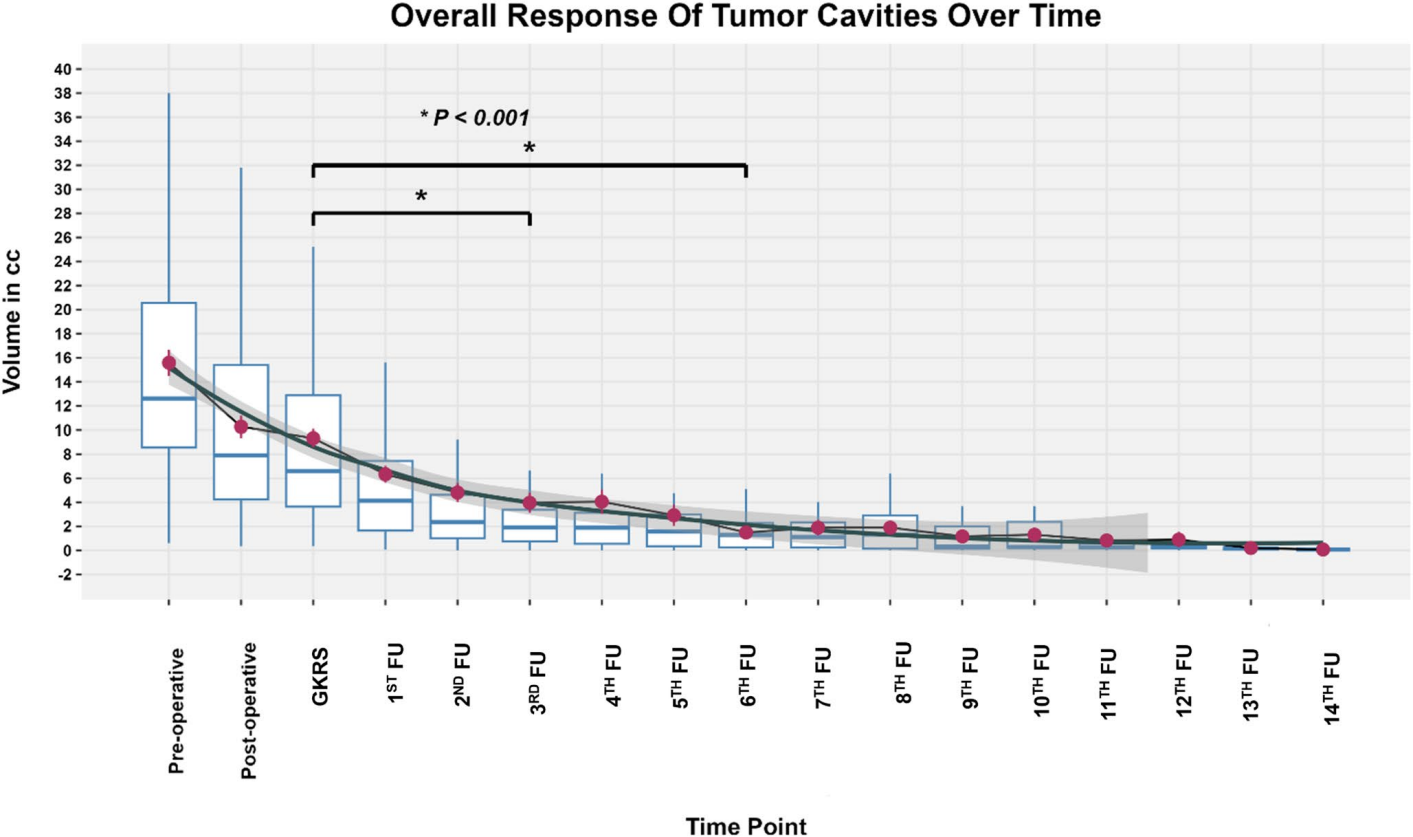
Longitudinal change in tumor cavity volume following gamma knife radiosurgery (GKRS).Boxplots display tumor cavity volume distributions across 17 timepoints, spanning preoperative baseline to the 14th post-treatment follow-up. Overlaid maroon point ranges represent mean ± standard error, and a LOESS-smoothed curve (dark slate gray) traces the cohort-level trajectory. Statistically significant reductions relative to the Gamma Knife Radiosurgery (GKRS) baseline are denoted by asterisks and significance bars. Notably, volumes at follow-ups 6 and 9 were significantly lower than at GKRS (*p* < 0.001). These differences were derived from a repeated-measures ANOVA with post hoc pairwise testing (FDR-adjusted). FU - Follow-up

Across serial MRIs, cavity volumes declined over time, with the largest reductions early and relative stabilization later (Fig. 2; Supplementary Figure S1). Observed follow-up timing (median months from GKRS with IQR) at each nominal follow-up is summarized in Supplementary Table S2.

In a linear mixed-effects model with ln-transformed cavity volume (ln[cc]) as the dependent variable and time from GKRS (months) as a continuous fixed effect, volume decreased significantly with time (Time (Months) β = −0.031; 95% CI − 0.044 to − 0.019; *p* < 0.001), corresponding to an estimated 3.1% reduction per month. A generalized additive model (GAM) confirmed nonlinearity (time spline edf = 3.78; F = 51.58; *p* < 0.001), consistent with rapid early involution followed by a plateau (Fig. 2). When time-from-GKRS was modeled categorically using prespecified follow-up windows (0–3, 3–6, 6–12, 12–24, > 24 months), results were concordant: repeated-measures ANOVA and a categorical-time mixed model both showed significant differences across follow-up timepoints (overall timepoint effect *p* < 0.001), with significant pairwise contrasts versus baseline across early-to-mid follow-up (Supplementary Table S3).

Negative linear values in Fig. 1 and Supplementary Figure S1 reflect cavity volumes < 1 cc rather than implausible “negative volumes.” Percent cavity volume change at the second follow-up (relative to GKRS planning volume) was not associated with overall survival (OS) on Cox regression (HR 0.84; *p* = 0.84).

### Local recurrence–free survival and local control

**Fig. 3:**
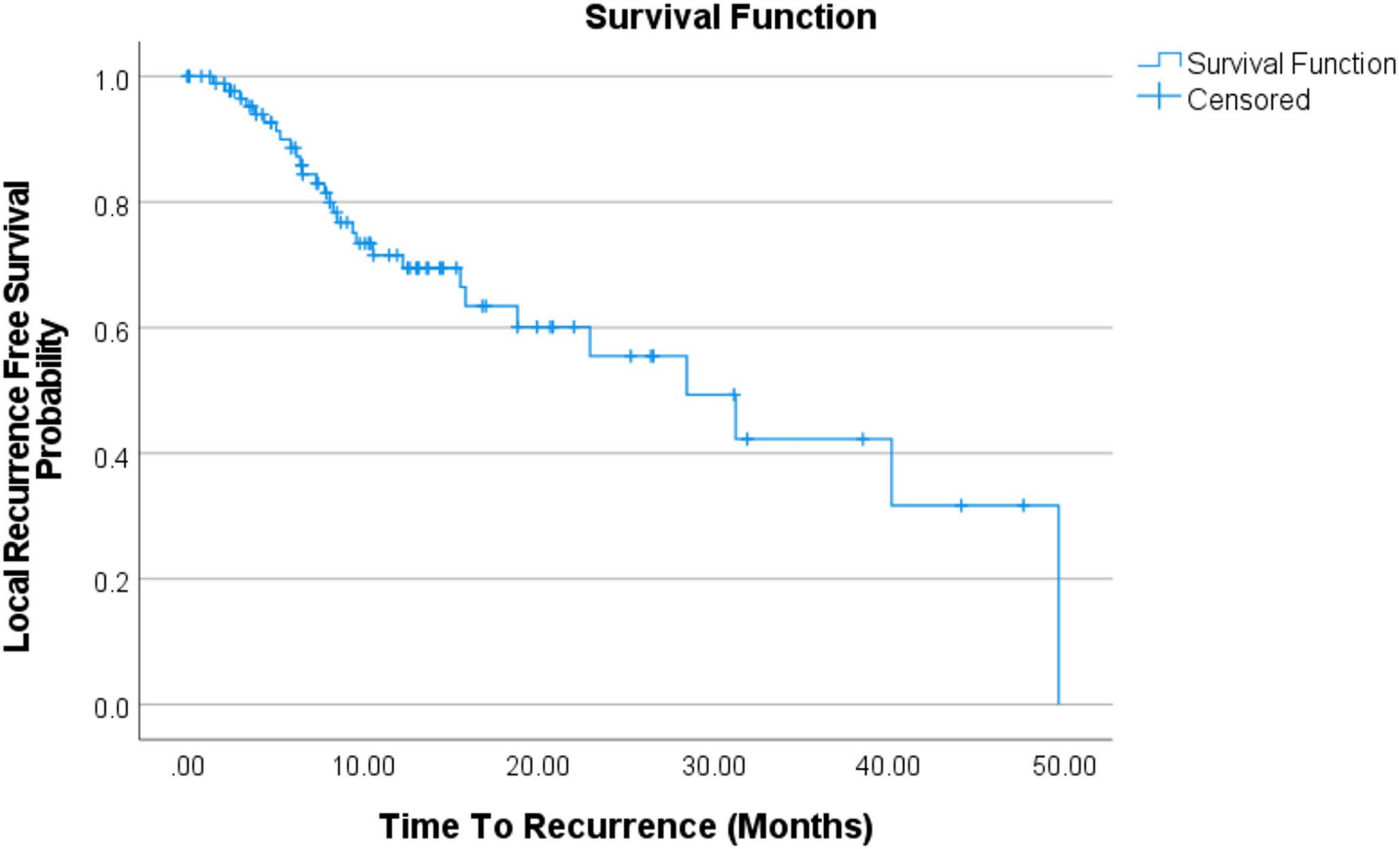
Cavity-level local control after GKRS. Kaplan–Meier curve showing local recurrence–free survival (local control) for treated resection cavities following Gamma Knife radiosurgery (GKRS). Time was measured from the date of GKRS to the first radiographic local recurrence. Cavities without documented recurrence were censored at the date of the last available imaging follow-up. Cavities from patients who died without documented local recurrence were censored at the date of death. Tick marks denote censored observations

Local recurrence was assessed at the cavity level. Of 98 cavities, 30 (30.6%) developed at least one local recurrence, and 12 (12%) experienced more than one recurrence episode during follow-up. Kaplan–Meier analysis showed a median local recurrence–free survival of 49.8 months (95% CI 23.0–76.5). Estimated local control at 6, 12, 24 and 36 was 89.7%, 74.5%, 62.5% and 54.9%, respectively (Fig. 3), indicating declining local control over time. Late estimates are provided in the supplement and should be interpreted cautiously due to attrition.

Cavities without recurrence were censored at the last intracranial imaging follow-up; cavities in patients who died without documented local recurrence were censored at the date of death. Late Kaplan–Meier estimates should be interpreted cautiously because radiographic surveillance decreased over time (Supplementary Table S1), reducing the at-risk set and widening uncertainty at later timepoints.

In exploratory analyses adjusted for baseline target volume (ln[VGK]), individual dosimetric parameters (prescribed dose, prescription isodose line, Dmin/Dmean/Dmax, coverage, PCI, GI, and PIV) were not significantly associated with LRFS. Treatment era was not associated with LRFS in sensitivity Cox models including ln(V_GK_), fractionation, and prescribed dose (2016–2020 vs. 2011–2015: HR 0.78, *p* = 0.63; 2021–2025 vs. 2011–2015: HR 0.92, *p* = 0.87).

### Volumetric trajectories by recurrence status

Cavity volumes declined over time in both recurrent and non-recurrent groups, with greater dispersion among cavities that later recurred (Supplementary Figure S2). In mixed-effects models, both recurrent and non-recurrent cavities showed significant decreases in volume over time (β = −1.46, *p* < 0.001, and β = −1.18, *p* < 0.001, respectively). Recurrent cavities had modestly larger baseline volumes (+ 2.34 cc), but the recurrence-by-time interaction was not significant (β = −0.282, *p* = 0.132), indicating that the rate of volumetric change did not distinguish cavities that later recurred from those that remained controlled.

### Tumor cavity dynamics and outcomes by primary cancer histology

**Fig. 4:**
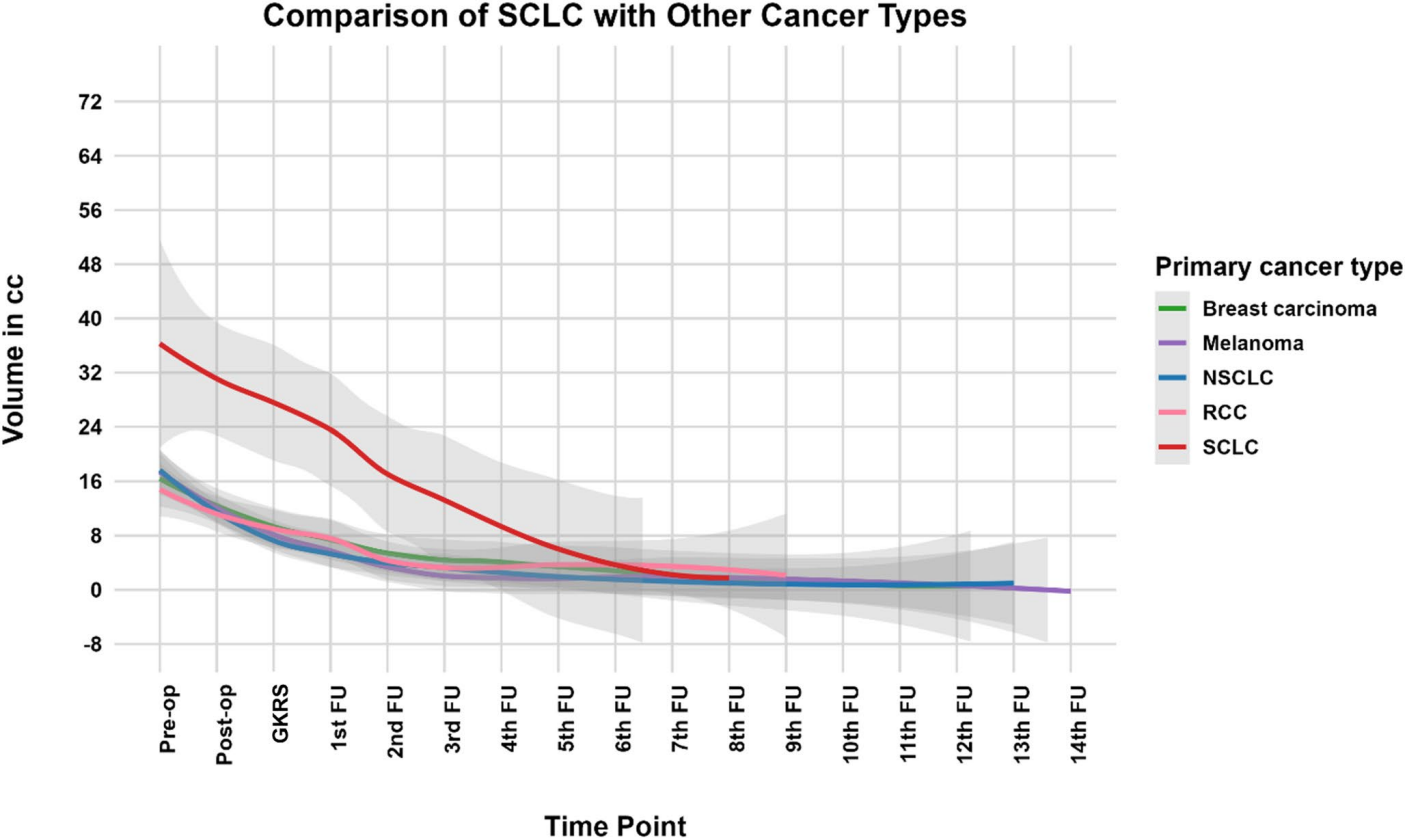
Tumor cavity volume trajectories stratified by primary cancer type. LOESS-smoothed curves with 95% confidence intervals show tumor cavity volume trajectories across 17 standardized timepoints, including preoperative, postoperative GKRS, and 14 follow-up intervals. Distinct patterns emerged across cancer types. Small cell lung cancer (SCLC) cavities began with the largest volumes and exhibited the steepest rate of reduction. In contrast, non-small cell lung cancer (NSCLC) and renal cell carcinoma (RCC) cavities contracted more slowly and more variably, while breast carcinoma and melanoma followed intermediate trajectories with relatively consistent declines and moderate inter-patient variability. GKRS - GammaKnife Radiosurgery. FU - Follow-up

Cavity volume trajectories were evaluated by primary histology across 17 standardized imaging timepoints (preoperative through 14 follow-ups). LOESS curves showed distinct patterns across non-small cell lung cancer (NSCLC; *n* = 37), small cell lung cancer (SCLC; *n* = 4), breast (*n* = 12), renal cell carcinoma (RCC; *n* = 12), and melanoma (*n* = 15), with SCLC exhibiting the largest baseline cavities and the steepest contraction (Fig. 4). In a linear mixed-effects model including timepoint, histology, and their interaction, cavity volume decreased over time overall (β = −1.22, *p* < 0.001). Relative to breast (reference), SCLC had a higher baseline volume (β = +21.35, *p* < 0.001) and a steeper decline over time (interaction β = −1.65, *p* = 0.0017); no other histology showed a significantly different slope.

Survival status proportions did not differ significantly by histology (Chi-square *p* = 0.177; Fisher’s exact *p* = 0.130). Overall survival duration also did not differ across histologies (ANOVA *p* = 0.839; Kruskal–Wallis *p* = 0.334), and Tukey HSD showed no significant pairwise contrasts (Supplementary Figure S3).

Given separation of OS distributions for RCC in across-histology summaries, RCC was compared with all other histologies (Fig. 4). RCC was associated with a lower hazard of death on Cox modeling (HR = 0.28, *p* = 0.034), and Kaplan–Meier analysis likewise showed longer OS for RCC versus non-RCC (log-rank *p* = 0.02; Supplementary Figure S4).

Cavity volume decreased with time in both survivors (*r* = − 0.53, *p* < 0.001) and patients who died (*r* = − 0.42, *p* < 0.001). Mixed-effects modeling showed that survivors had lower overall cavity volumes (β = −5.15, *p* = 0.0048) and a less steep decline over time (interaction β = +0.37, *p* = 0.034).

### Functional status tracks with cavity burden and predicts survival

Performance status predicted OS at both evaluated timepoints (Supplementary Figure S5). Favorable status (KPS ≥ 70 or ECOG ≤ 1) at craniotomy was associated with improved survival (HR = 0.41; 95% CI 0.19–0.92; *p* = 0.029), and this association remained significant at GKRS (HR = 0.45; 95% CI 0.26–0.78; *p* = 0.0049). At GKRS, KPS was inversely correlated with cavity volume (Pearson *r* = − 0.34, *p* = 0.0021), indicating worse function among patients with larger cavities (Supplementary Figure S6).

In an era-adjusted Cox model for OS measured from GKRS, KPS at GKRS remained associated with improved survival (HR 0.95 per 1-point increase, *p* < 0.001) and older age was associated with inferior survival (HR 1.06 per year, *p* = 0.001); treatment era was not independently associated with OS (2016–2020 vs. 2011–2015: HR 0.58, *p* = 0.13; 2021–2025 vs. 2011–2015: HR 0.66, *p* = 0.30).

### Treatment modality, preoperative volume, and outcomes

**Fig. 5:**
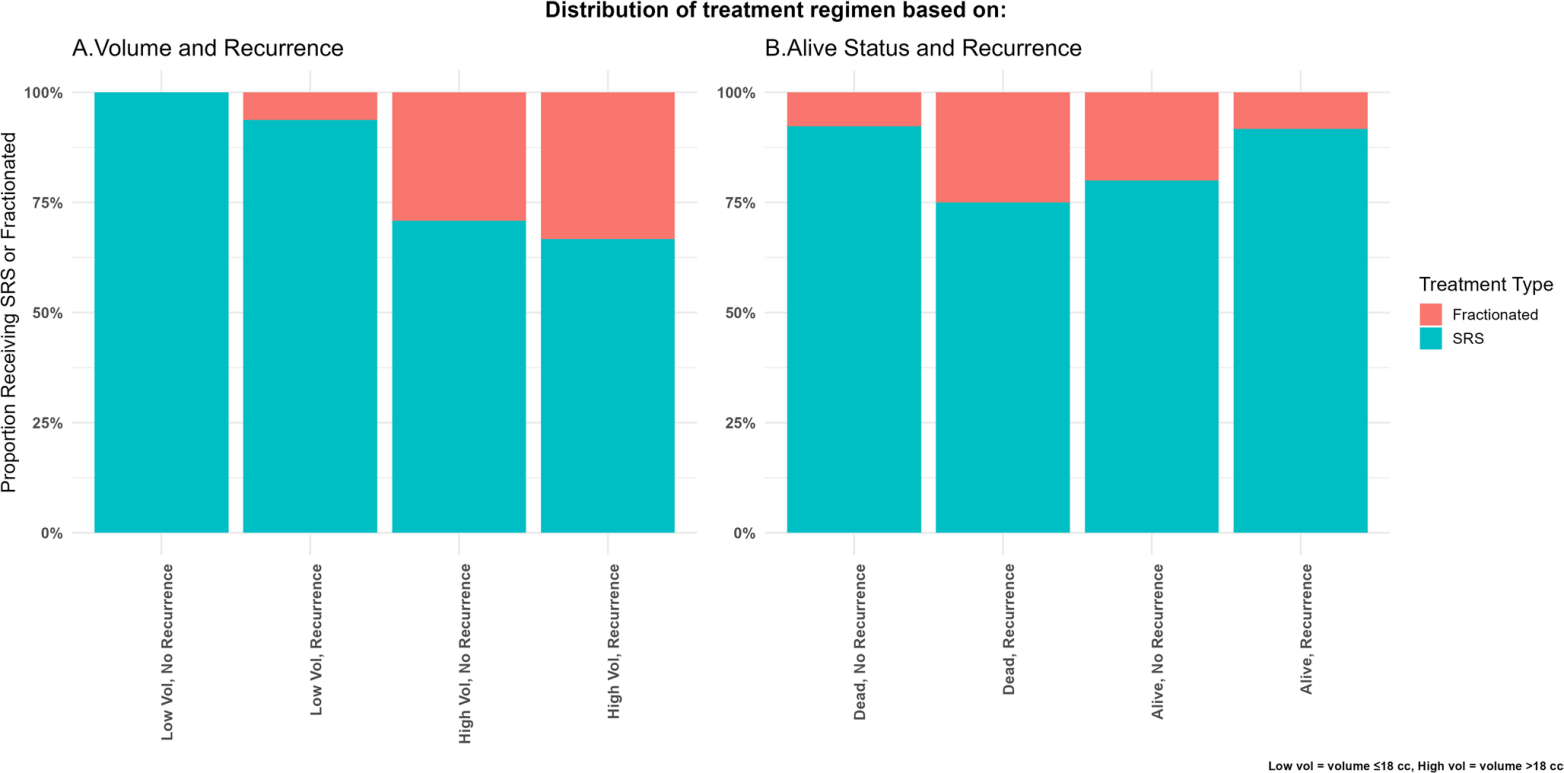
Clinical outcomes stratified by treatment modality and tumor volume. **A**. Recurrence rates stratified by preoperative tumor volume (< 18 cc vs. ≥18 cc) and Gamma Knife radiosurgery type (sfGKRS vs. hfGKRS). **B. **Combined recurrence and survival status stratified by treatment modality

The relationship between radiosurgical strategy (sfGKRS vs. hfGKRS), preoperative tumor volume, and outcomes is summarized in Fig. 5. Treatment type was associated with the combined stratification of preoperative volume and recurrence status (Fisher’s exact *p* = 0.0004) but not with recurrence when evaluated alongside survival status (Fisher’s exact *p* = 0.26).

Within preoperative volume strata, recurrence rates did not differ between sfGKRS and hfGKRS for tumors < 18 cc (*p* = 0.31) or ≥ 18 cc (*p* = 1.00). In multivariable logistic regression, no significant interaction between treatment modality and preoperative volume was identified, and fractionation strategy was not an independent predictor of recurrence.

## Discussion

In this single-institution cohort of resected brain metastasis cavities treated with adjuvant GKRS, we observed three clinically relevant themes. First, postoperative cavities demonstrated a reproducible, time-dependent involution pattern with rapid early contraction followed by a decelerating trajectory and a later plateau, consistent with non-linear remodeling. Second, local failure remained clinically meaningful: 30% of cavities recurred. Third, patient-level status and disease context influenced outcomes - performance status was inversely correlated with cavity burden and independently predicted OS, consistent with established prognostic frameworks in brain metastases in which KPS is a dominant determinant across histologies [16]. Histology-associated differences, including favorable RCC survival in this dataset, further support that intracranial outcomes increasingly reflect systemic disease biology and treatment era effects [17, 18]. We adjusted outcome models for treatment era to partially mitigate platform and systemic-therapy evolution.

The temporal behavior of postoperative cavities has direct implications for radiosurgical planning because the resection bed is not static. Cavity volumes decreased significantly with time in longitudinal mixed-effects modeling, and non-linearity was supported by spline-based modeling, demonstrating early involution with later stabilization (Figs. 1 and 2; Supplementary Figure S1). Although stereotactic MRI at the time of GKRS is standard, our data quantify the magnitude and nonlinearity of postoperative cavity remodeling across clinically used follow-up windows and demonstrate that early postoperative cavity geometry is an unreliable surrogate for the GKRS planning target. This is most relevant for larger cavities and those near OAR constraints, where modest geometric shifts can meaningfully alter plan feasibility and hotspot distribution. This aligns with prior postoperative cavity dynamics literature emphasizing moving target behavior and the importance of contouring on contemporary imaging rather than extrapolating from earlier postoperative scans [12, 13, 19]. 

Importantly, our supplementary follow-up timing table contextualizes nominal timepoints with real-world imaging cadence and illustrates attrition at later follow-ups, which is relevant to interpreting longitudinal trends and counseling regarding surveillance intensity (Supplementary Tables S1–S2).

Despite adjuvant GKRS, local recurrence occurred in nearly one-third of cavities, and local control declined over time (Fig. 3). This is consistent with the broader postoperative GKRS literature, which reports a persistent failure risk of 20 to 40% range [20]. Cavity involution likely reflects postoperative brain re-expansion and dural settling rather than a radiation-induced phenomenon. Consistent with this, volumetric kinetics did not discriminate recurrent from controlled cavities, suggesting that local failure is driven more by anatomic risk regions and dosimetric coverage than by bulk volumetric regression.

Plan-level parameters allowed us to contextualize failure rates - hypofractionation was used for larger targets and was associated with higher prescribed dose and higher Dmax/Dmean, reflecting expected selection for larger or more complex cavities. Despite these differences in planning context, we did not identify a significant association between individual dosimetric parameters and LRFS after accounting for baseline target volume, suggesting that within standard institutional practice ranges, cavity kinetics and gross dosimetric summaries alone may be insufficient to explain local failure. Local failure is also understood to be multifactorial, reflecting factors beyond size alone, including residual microscopic disease, cavity surface complexity and irregularity, proximity to the dura or ventricle, resection tract involvement, and adequacy of dosimetric coverage of high-risk regions [21–24]. In our cohort, volumetric trajectories declined in both recurrent and non-recurrent cavities, and the recurrence-by-time interaction was not statistically significant, indicating that cavity kinetics alone do not reliably discriminate between recurrent and non-recurrent cavities. This supports the need for recurrence stratification approaches that integrate anatomic and dosimetric features rather than relying on early volumetric regression as a standalone biomarker.

Late Kaplan–Meier estimates should be interpreted with caution because the at-risk set diminishes with decreasing radiographic surveillance over time (Supplementary Table S1), widening uncertainty around late estimates. Within preoperative volume strata, recurrence rates did not differ significantly between sfGKRS and hfGKRS. The fractionation strategy was not an independent predictor of recurrence in multivariable modeling. Clinically, this is consistent with the prevailing practice of selecting fractionation primarily to satisfy OAR constraints and manage toxicity risk in large, irregular, or eloquently located cavities, rather than expecting fractionation alone to overcome biology-driven local failure [7, 8, 25, 26]. Functional status was closely linked to radiographic burden and outcomes: lower KPS at GKRS correlated with larger cavity volumes, and performance status at both craniotomy and GKRS predicted OS (Supplementary Figures S5–S6). These findings reinforce the clinical relevance of neurologic reserve in postoperative decision-making and counseling, and they align with established prognostic frameworks in brain metastases where performance status is a dominant determinant of outcomes [16]. 

### Limitations

This retrospective single-institution study is subject to selection effects and residual confounding. Imaging follow-up was nonuniform and decreased over time, limiting the precision of late Kaplan–Meier estimates and long-term kinetic inference, although early and mid-term trends were consistent across scans. Treatment heterogeneity (fractionation strategy, timing of GKRS, and evolving systemic therapy) reflects real-world practice but complicates the isolation of individual effects. Some histology subgroups were small, and volumetric segmentation, despite a standardized workflow, remains susceptible to variability in irregular cavities and postoperative change. Post-GKRS treatment effect (including radionecrosis) can alter enhancement patterns and may affect apparent cavity boundaries despite adjudication with standard MRI and CCA; this may introduce measurement variability in longitudinal volumes. Although era was not independently associated with LRFS or OS in sensitivity models, residual confounding from evolving systemic therapies with intracranial activity remains possible.

## Conclusion

Postresection cavities treated with adjuvant GKRS demonstrate rapid early involution followed by relative stabilization, and volumetric kinetics alone did not distinguish cavities that recurred from those that remained controlled. Local failure remains a persistent risk over time, underscoring the need for ongoing surveillance and continued optimization of target design and coverage of high-risk regions. Interpretation of local failure requires explicit consideration of target definition and dosimetric coverage of high-risk regions. 

## Supplementary Information

Below is the link to the electronic supplementary material.

Supplementary Material 1

## Data Availability

The datasets generated during and/or analyzed during the current study are available from the corresponding author on reasonable request.

## Abbreviations

CCA: Contrast Clearance Analysis
CSF: Cerebrospinal fluid
ECOG: Eastern Cooperative Oncology Group
GKRS: Gamma Knife Radiosurgery
hfGKRS: Hypofractionated Gamma Knife Radiosurgery
KPS: Karnofsky Performance Status
LMD: Leptomeningeal Disease
LRFS: Local recurrence–free survival
OS: Overall survival
sfGKRS: Single-fraction Gamma Knife Radiosurgery
SRS: Stereotactic Radiosurgery
WBRT: Whole brain radiotherapy
