# Supplementary material for: Longitudinal remodeling of brain metastasis resection cavities after adjuvant gamma knife radiosurgery
Source: J Neurooncol. 2026 Apr 28;177(3):129. doi: 10.1007/s11060-026-05567-7 (PMC13124882; doi:10.1007/s11060-026-05567-7)
Supplement: Supplementary file 1 — Supplementary Material 1 [file 11060_2026_5567_MOESM1_ESM.docx]

**Longitudinal Remodeling of Brain Metastasis Resection Cavities After Adjuvant Gamma Knife Radiosurgery**

**Supplemental Material**

**Table S1**

| **Time window post-GKRS** | **N cavities with ≥1 MRI in window** |
| --- | --- |
| 0–3 months | 102 |
| 3–6 months | 68 |
| 6–12 months | 63 |
| 12–24 months | 32 |
| >24 months | 15 |

**Supplementary Table S1.** Imaging availability after GKRS. Imaging availability is summarized as the number of resection cavities with at least one post-treatment MRI within each time window. Counts are not mutually exclusive because a given cavity may contribute imaging to more than one window. In total, 98 cavities had at least one MRI within these windows.

**Table S2**

| **Follow-up** | **N** | **Median months post-GKRS (IQR)** |
| --- | --- | --- |
| FU1 | 93 | 1.9 (0.7) |
| FU2 | 77 | 4.8 (1.8) |
| FU3 | 63 | 7.4 (2.9) |
| FU4 | 46 | 10.5 (3.6) |
| FU5 | 34 | 13.6 (5.2) |
| FU6 | 23 | 17.6 (4.1) |
| FU7 | 23 | 20.8 (6.4) |
| FU8 | 14 | 23.8 (10.8) |
| FU9 | 10 | 27.1 (17.2) |
| FU10 | 7 | 31.9 (19.1) |
| FU11 | 6 | 35.8 (21.3) |
| FU12 | 6 | 40.1 (20.7) |
| FU13 | 5 | 37.4 (23.1) |
| FU14 | 1 | — |

**Supplementary Table S2.** Follow-up timepoints after GKRS. Follow-up (FU) timepoints are summarized as median months from GKRS, with interquartile range (IQR). N indicates the number of cavities with evaluable MRI at each follow-up. FU14 had only one observation; thus, dispersion statistics are not shown. FU14 has N=1, so a median/IQR is not meaningful; FU13–FU14 should generally be interpreted cautiously due to sparse observations.

**Table S3**

| **Timepoint vs GKRS baseline** | **Mean difference (cc)** | **95% CI** | **p-value (adjusted*)** |
| --- | --- | --- | --- |
| Second follow-up | 5.79 | 1.12 to 10.46 | 0.0023 |
| Third follow-up | 6.64 | 1.66 to 11.61 | 0.0005 |
| Fourth follow-up | 6.55 | 1.11 to 11.98 | 0.0038 |
| Fifth follow-up | 7.68 | 1.58 to 13.77 | 0.0017 |
| Sixth follow-up | 9.11 | 2.14 to 16.08 | 0.0008 |
| Seventh follow-up | 8.70 | 1.61 to 15.80 | 0.0027 |

**Supplementary Table S3**. Pairwise contrasts in cavity volume versus GKRS baseline.

(Estimates are shown as absolute mean difference in volume [cc], baseline − follow-up; positive values indicate smaller cavities at follow-up.) *These p-values are FDR-adjusted (False Discovery Rate).

**Figure S1**


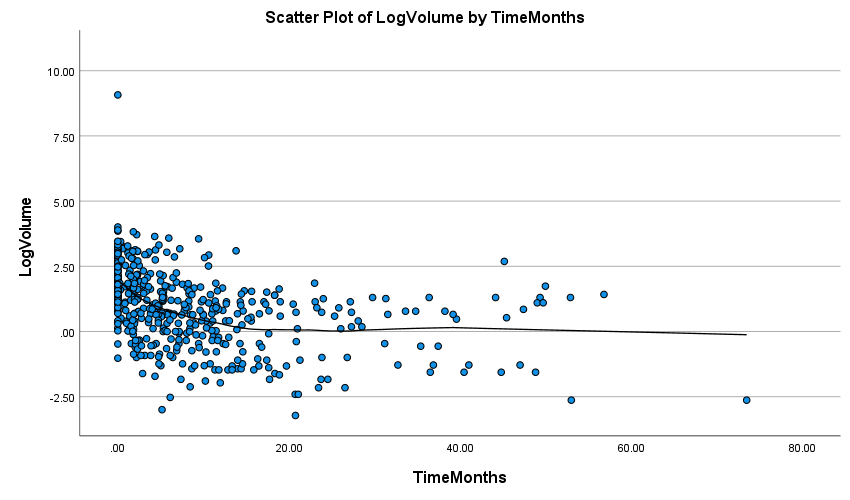
**Supplemental Figure S1**. Longitudinal resection cavity volume after GKRS. Scatterplot of natural log–transformed cavity volume (ln[cc]) versus time in months after GKRS. A LOESS smooth is overlaid to illustrate the overall temporal trajectory of cavity involution; ln values may be negative for volumes <1 cc.

**Figure S2**


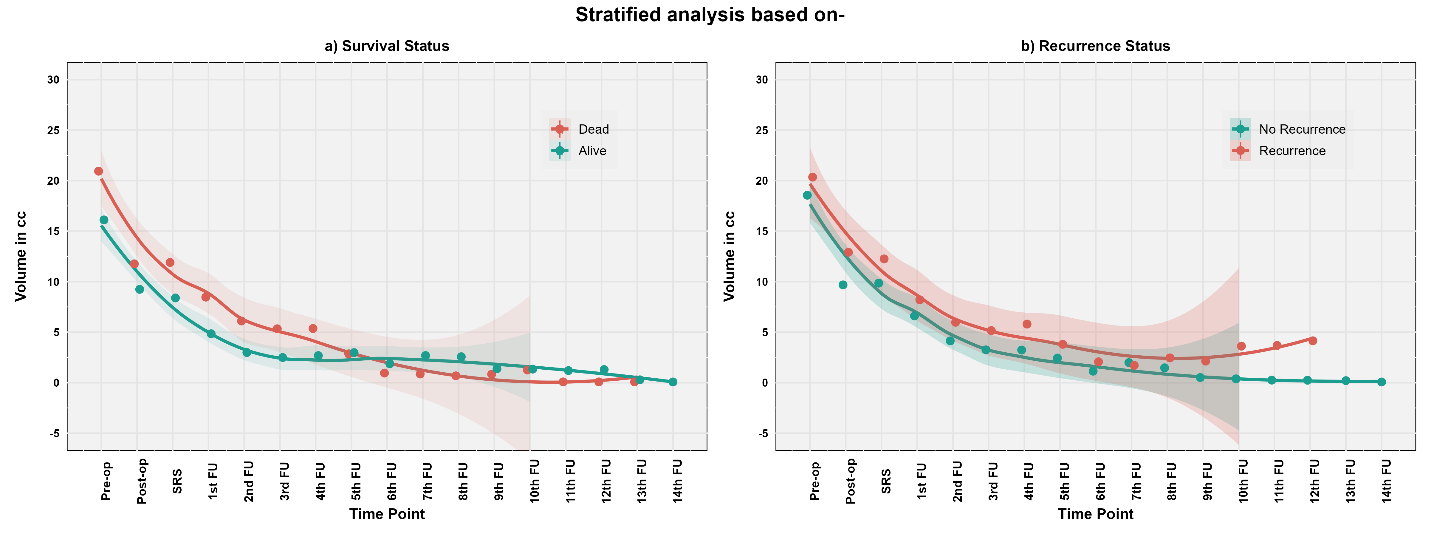


***Supplementary Figure S2: Longitudinal Volumetric Response of Tumor Cavities Stratified by Clinical Outcomes.***
***(a)*** *Tumor cavity volumes over time, stratified by survival status.* ***(b)*** *Tumor cavity volumes over time, stratified by recurrence status. Each panel shows the mean volume at each time point (± standard error) and a smoothed LOESS trajectory with shaded 95% confidence intervals (up to the tenth follow-up). In both comparisons, all patient groups showed volumetric reduction over time following GKRS. However, subtle differences emerged by outcome: patients who survived or remained recurrence-free exhibited a steadier, more gradual contraction, whereas those who died or recurred had larger baseline volumes and more pronounced early shrinkage. Visual differences were observed; however, formal interaction testing did not support differential volumetric slopes by outcome.*

**Figure S3**

**
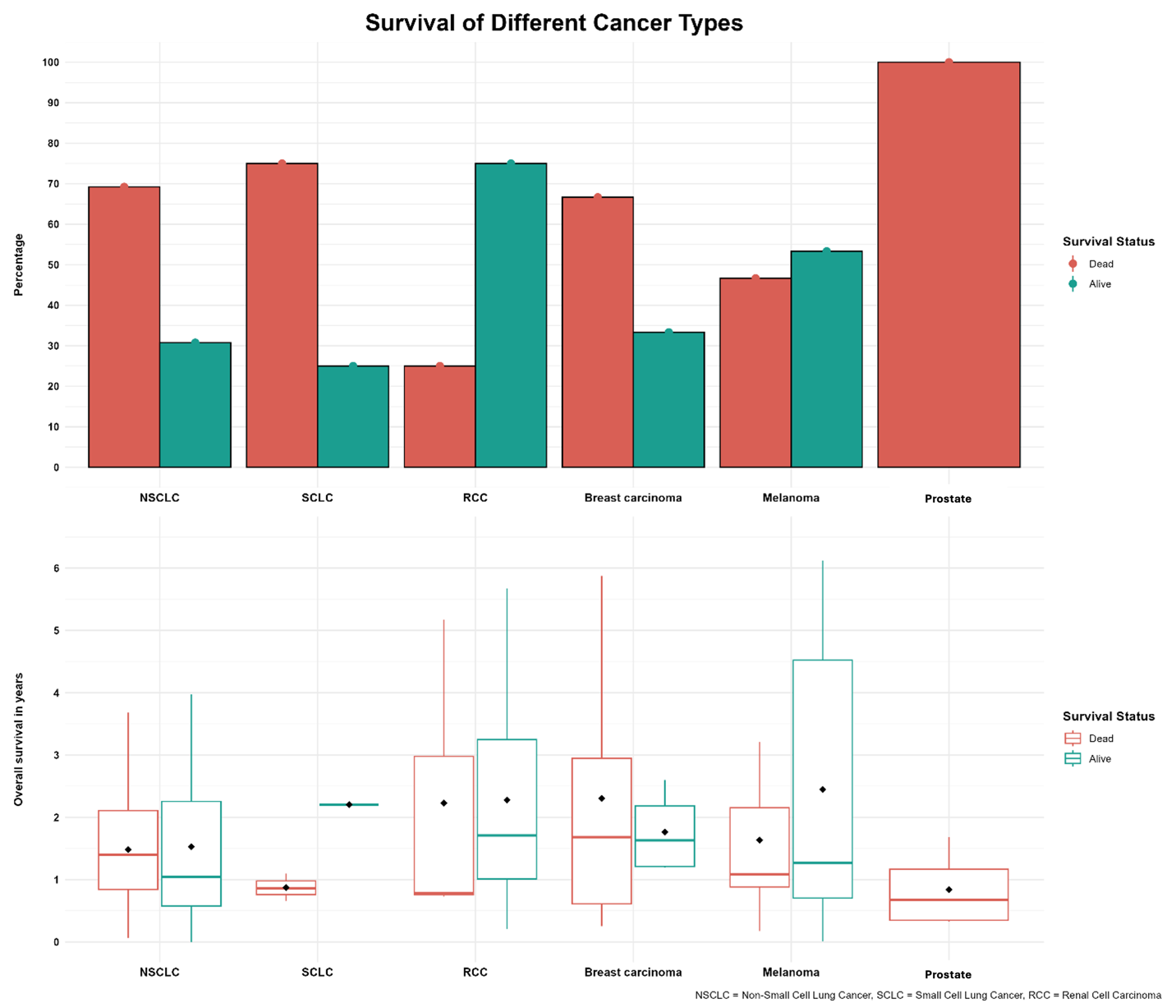
**

**Supplementary Figure S3**. ***Overall survival stratified by primary cancer type.*** ***(Top):*** *Proportion of surviving and patients who died at study completion.* ***(Bottom):*** *Distribution of overall survival time in years. No statistically significant differences were observed across groups (Chi-square and ANOVA, both not significant).*

**Figure S4**


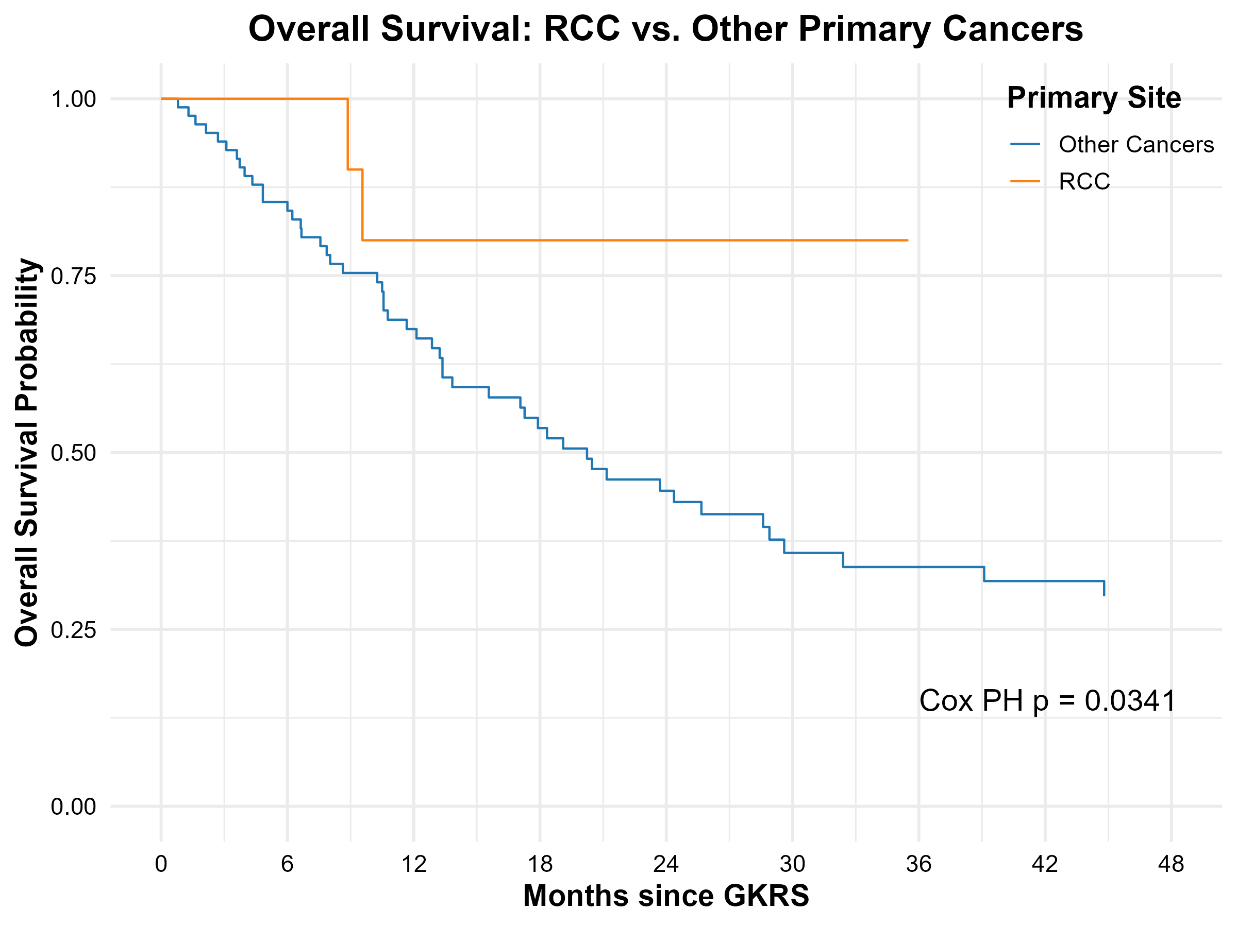


**Supplementary Figure S4*.*** ***Overall Survival in RCC vs. Other Histologies***

*Kaplan–Meier survival curve comparing patients with brain metastases from renal cell carcinoma (RCC) with those from other primary cancers. RCC patients had significantly improved overall survival. A Cox proportional hazards model confirmed that RCC was independently associated with a lower mortality risk (p = 0.034).*

**Figure S5**

***
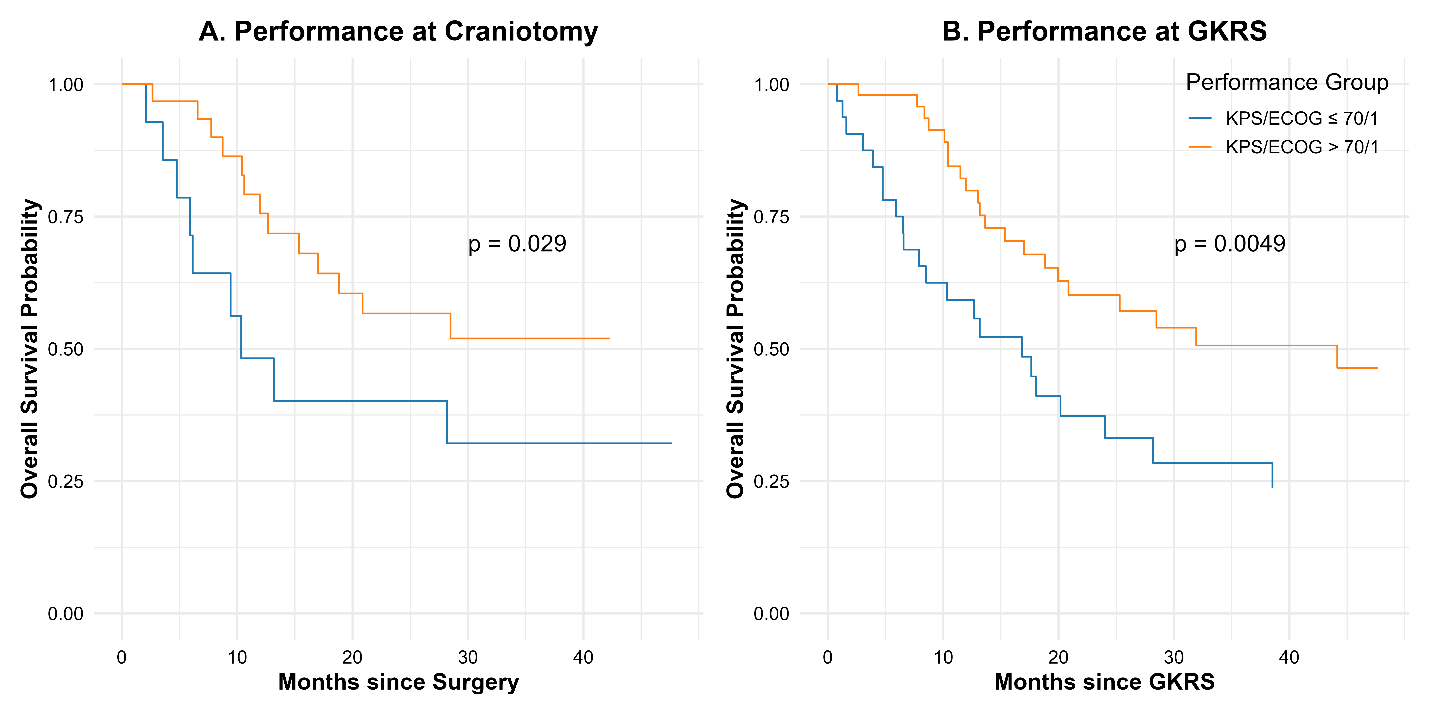
*Supplementary Figure S5***.* ***Kaplan-Meier Curves for Overall Survival by Performance Status*** *(A) Kaplan-Meier survival curves stratified by Karnofsky Performance Score (KPS) and ECOG status at the time of craniotomy. (B) Corresponding curves stratified by performance status at the time of Gamma Knife Radiosurgery (GKRS).*

**Figure S6**


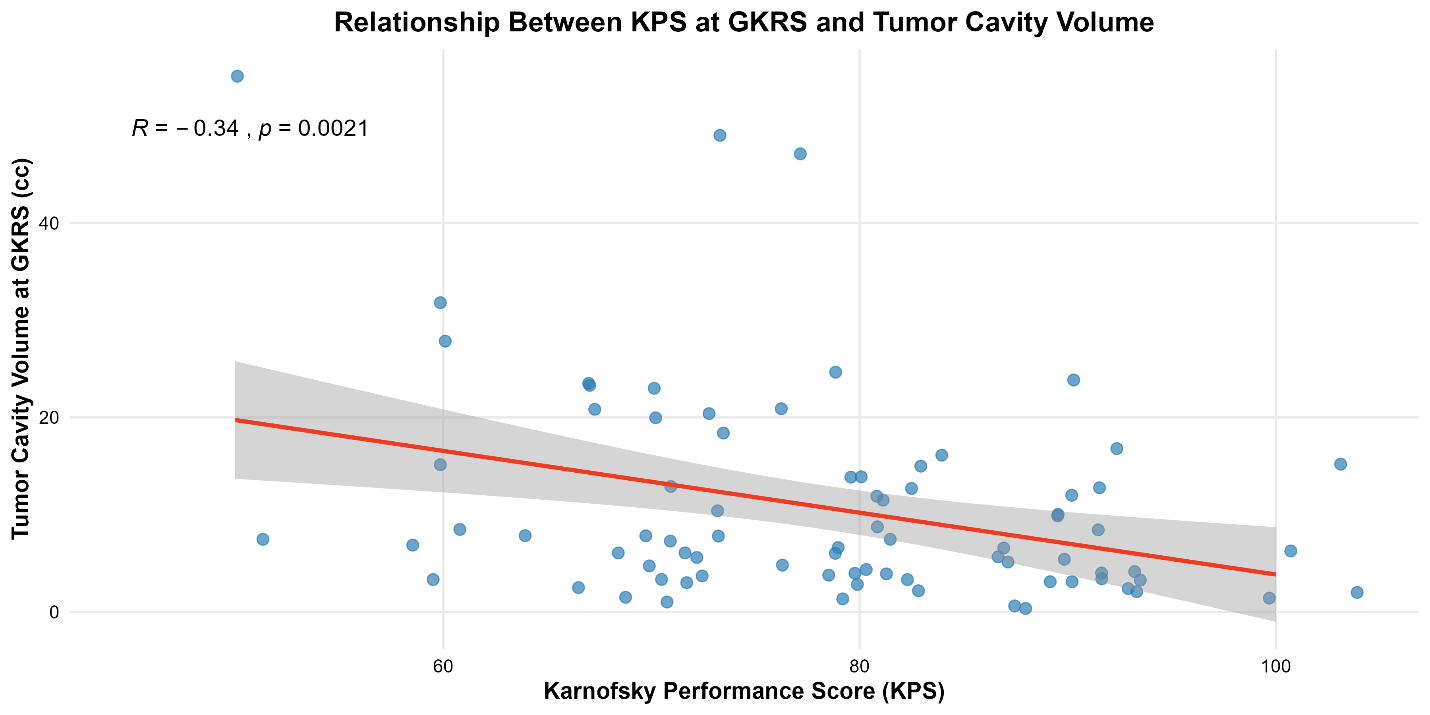


**Supplementary Figure S6*.*** ***Relationship Between Karnofsky Performance Score and Tumor Cavity Volume.*** *Scatter plot showing the correlation between Karnofsky Performance Score (KPS) at the time of Gamma Knife Radiosurgery (GKRS) and tumor cavity volume. A significant inverse association was observed (Pearson’s r = –0.34, p = 0.0021), indicating that patients with larger postoperative cavities tended to have lower functional status.*
